# The Outcome of Antifungal Prophylaxis with Posaconazole in Patients with Acute Myeloid Leukemia: A Single-Center Study

**DOI:** 10.4274/tjh.2017.0430

**Published:** 2018-11-13

**Authors:** Vildan Özkocaman, Fahir Özkalemkaş, Serdar Seyhan, Beyza Ener, Ahmet Ursavaş, Tuba Ersal, Esra Kazak, Ezgi Demirdöğen, Reşit Mıstık, Halis Akalın

**Affiliations:** 1 Uludağ University Faculty of Medicine, Department of Internal Medicine, Division of Hematology, Bursa, Turkey; 2 Uludağ University Faculty of Medicine, Department of Medical Microbiology, Bursa, Turkey; 3 Uludağ University Faculty of Medicine, Department of Chest Disease and Tuberculosis, Bursa, Turkey; 4 Uludağ University Faculty of Medicine, Department of Infectious Disease and Clinical Microbiology, Bursa, Turkey

**Keywords:** Acute myeloid leukemia, Invasive fungal infections, Antifungal prophylaxis, Posaconazole

## Abstract

**Objective:**

Invasive fungal infections (IFIs) are a significant cause of morbidity and mortality among neutropenic patients undergoing chemotherapy for acute myeloid leukemia (AML) and stem cell transplantation. The aim of this study was to evaluate the real-life impact of posaconazole prophylaxis.

**Materials and Methods:**

Eighty-four adult patients were included with AML under remission induction chemotherapy and posaconazole prophylaxis. The 34 patients in the control group did not receive primary antifungal prophylaxis. The period between June 2006 and January 2009, when antifungal prophylaxis was not administered (control group), was retrospectively compared to the period between December 2010 and May 2012 when primary oral posaconazole prophylaxis was administered in similar conditions (posaconazole group) according to the use of antifungal agents for treatment, breakthrough infections, galactomannan performance, and the necessity for performing bronchoalveolar lavage (BAL) procedures.

**Results:**

The two groups were compared according to the use of antifungal agents; progression to a different antifungal agent was found in 34/34 patients (100%) in the control group and in 9/84 patients (11%) in the posaconazole group (p<0.001). There were four breakthrough IFIs (4/84, 4.8%) in the posaconazole group and 34 IFIs in the control group (p<0.001). In addition, 15/34 patients (44%) in the control group required BAL compared to 11/84 patients (13%) in the posaconazole group (p<0.001). Posaconazole treatment was discontinued within 7-14 days in 7/84 patients (8.3%) due to poor oral compliance related to mucositis after chemotherapy.

**Conclusion:**

Posaconazole appears to be effective and well-tolerated protection against IFIs for AML patients.

## Abstract (tr)

**Amaç:**

İnvaziv fungal enfeksiyonlar (İFE) akut myeloid lösemili (AML) ve kök hücre nakli yapılan hastalarda önemli bir mortalite ve morbidite nedenidir. Bu çalışmanın amacı posakonazol profilaksisinin gerçek yaşamdaki etkisini değerlendirmektir.

**Gereç ve Yöntemler:**

AML ve remisyon indüksiyon kemoterapisi alan ve posakonazol profilaksisi uygulanan 84 erişkin hasta çalışmaya dahil edildi. Kontrol grubunda primer antifungal profilaksi almayan 34 hasta dahil edildi. Haziran 2006 ile Ocak 2009 tarihleri arası primer oral posakonazol profilaksisi almayan (kontrol grubu) ile Aralık 2010 ile Mayıs 2012 arası primer oral posakonazol profilaksisi (posakonazol grubu) uygulanan hastaları geriye dönük olarak; tedavi için antifungal ajan kullanımı, tedavi altında (breakthrough) enfeksiyonlar, galaktomannan performansı ve bronko-alveolar lavaj (BAL) gerekliliği gibi benzer durumlar için karşılaştırdık.

**Bulgular:**

İki grup antifungal ajan kullanımına göre karşılaştırıldığında farklı antifungal ajana geçiş kontrol grubunda 34/34 (%100) idi ve posakonazol grubunda bu oran 9/84 (%11) bulundu (p<0,001). Posakonazol grubunda 4 tedavi altında (breakthrough) IFE (4/84, %4,8) ve kontrol grubunda ise 34 İFE vardı (p<0,001). İlaveten kontrol grubunda BAL gereken hasta 15/34 (%44) iken, posakonazol grubunda BAL gerekliliği 11/84 (%13) bulundu (p<0,001). Posakonazol tedavisi hastaların 7/84’ünde (%8,3) kemoterapi sonrası mukozite bağlı oral alım bozukluğu nedeniyle 7-14 gün içinde kesilmişti.

**Sonuç:**

Posakonazol AML’li hastaların invaziv fungal enfeksiyonlarına karşı korumada etkili ve iyi tolere ediliyor görünmektedir.

## Introduction

Invasive fungal infections (IFIs) are a significant cause of morbidity and mortality among neutropenic patients undergoing chemotherapy for acute myeloid leukemia (AML) and stem cell transplantation. Antifungal prophylaxis is an important aspect of treatment because these infections are often difficult to diagnose due to their lack of specific clinical features [1,2]. The use of mold-specific prophylaxis has increased in recent years, particularly in AML patients, because of the high mortality rate of IFIs [1,2,3]. Posaconazole has been recommended as the drug of choice for AML patients undergoing induction chemotherapy based on the results of randomized controlled trials [4,5,6,7,8].

The aim of this study was to evaluate the real-life impact of posaconazole prophylaxis. Patients under posaconazole prophylaxis who were followed from 2010 to 2012 were compared with historical control patients without posaconazole prophylaxis who were followed from 2006 to 2009 in similar conditions according to the use of antifungal agents for treatment, breakthrough infections, galactomannan (GM) performance, and the requirement for bronchoalveolar lavage (BAL) procedures.

## Materials and Methods

A retrospective single-center study on primary prophylaxis with posaconazole was conducted in the Department of Hematology at the Uludağ University Hospital, a tertiary care hospital with 900 beds accredited by the Joint Commission International. Patients had to meet the following inclusion criteria to be eligible for this study: 18 years or older age, AML diagnosis, under remission induction or salvage chemotherapy, and under treatment at the hospital between December 2010 and May 2012. There were no patients with myelodysplastic syndrome (MDS) in either group. This retrospective study (number 2012-13/1; 19 June 2012) was approved by the local ethics committee for data collection.

Eighty-four adult patients were included with AML under remission induction chemotherapy and posaconazole prophylaxis who were followed from December 2010 to May 2012. In accordance with the indications for high-risk episodes, prophylactic treatment was started 24 h after the last day of chemotherapy and continued until neutrophil levels recovered to >0.5x10^9^/L. Posaconazole (200 mg, oral suspension) was given orally three times daily. Thirty-four patients undergoing remission induction chemotherapy for AML who were not under posaconazole prophylaxis and who were followed from 2006 to 2009 were included as a control group. The control group did not receive any antifungal prophylaxis. The posaconazole-treated patients were compared with the control group according to the use of antifungal treatment, breakthrough infections, GM performance, and the need for BAL.

In 2008, the Infectious Disease Society of America (IDSA) recommended posaconazole for antifungal prophylaxis in hematopoietic stem cell transplantation recipients with graft-versus-host disease and for neutropenic patients with AML or MDS [9]. The protocol for treating febrile neutropenia was based on the clinical practice guidelines for the use of antimicrobial agents in neutropenic patients with cancer that were introduced by the IDSA in 2002 and updated in 2010.

According to the National Institute of Allergy and Infectious Diseases Mycoses Study Group (EORTC/MSG) criteria, the clinical decision to replace prophylaxis with intravenous antifungal therapy was based in all cases on an individualized clinical judgment. This decision took into account the patient’s general condition, the patient’s signs and symptoms, the test results, and the patient’s treatment compliance. The incidence and reason for early discontinuation of prophylaxis and the cause of death were recorded in all applicable cases.

*Aspergillus* galactomannan tests (Platelia* Aspergillus*; Bio-Rad Laboratories, Marnes-la-Coquette, France) were performed for the BAL and bronchial lavage specimens according to the manufacturer’s instructions [10,11]. The patients were followed by high-resolution pulmonary computerized tomography (CT), serum GM, BAL, and BAL GM during the course of antifungal treatment in our clinic to refine the overall treatment strategy. The levels of GM in serum were measured twice a week for all of the patients. The serum GM test results were available within 2 days and were considered to be positive if the optical index was >0.7 in one sample or ≥0.5 in two consecutive samples. The BAL results for GM were considered to be positive if the BAL GM was ≥1.5.

A high-resolution pulmonary CT scan was performed between days 5 and 7 of febrile neutropenia or in the case of clinical deterioration.

These data were used to direct the treatment strategy and guide preemptive antifungal therapy at the study center. A multidisciplinary approach was used to make treatment decisions; a hematologist, an infectious disease specialist, a medical microbiologist, and a pulmonologist were consulted. Special attention was given to clinical, radiographic, and microbiological signs of infection; the duration of neutropenia; and the antimicrobial therapy. There was no difference in the daily diagnostic and therapeutic approaches and the physical and environmental conditions during the entire period. There were no HEPA filters or constructional changes in our inpatient clinic in either period. If there was evidence of invasive fungal disease, it was classified according to the 2008 revised EORTC/MSG criteria as ‘possible’, ‘probable’, or ‘proven’ IFI [12,13].

Breakthrough IFI was considered if IFI occurred four or more days after the initiation of primary antifungal prophylaxis with posaconazole [14].

### Statistical Analysis

Statistical analyses were performed with SPSS 20.0 for Windows (IBM Corp., Armonk, NY, USA). The data are expressed as means ± standard deviation and were compared as follows: continuous variables were compared using the Mann-Whitney U test, categorical variables were compared using the chi-square test, and p<0.05 was considered significant. Data analyses were performed using Fisher’s exact test and chi-square analysis.

## Results

The patients’ characteristics are summarized in Table 1. The average age, distribution of sex, medical history, and underlying disease status were similar in the two groups. In the posaconazole group, there were 84 patients with AML. The median age of the patients was 49.5 years (min-max: 20-71) and 54% of the patients in the posaconazole group were female. Of these 84 patients, 68 had received remission induction chemotherapy for a newly diagnosed disease and 16 had received salvage chemotherapy for relapsed disease. The median duration of primary posaconazole prophylaxis was 28 days (min-max: 7-60) in the posaconazole group and there was no toxicity related to posaconazole treatment. Posaconazole treatment was discontinued within 7-14 days in seven of 84 patients (8.3%) due to poor oral compliance related to mucositis after chemotherapy (Table 2). Two of these patients developed IFIs (1 possible, 1 probable). In addition to that, two patients without mucositis were diagnosed with breakthrough IFIs (1 possible, 1 probable) during posaconazole prophylaxis and their antifungal treatment was changed. There was no breakthrough IFI in 75 patients who completed posaconazole prophylaxis. Totally, there were four breakthrough infections in the posaconazole prophylaxis group (4/84, 4.8%). There were 28 possible and 6 probable IFIs in the control patients.

Antifungal therapy was given to seven of these patients. The antifungal drugs used were conventional amphotericin B, itraconazole, liposomal amphotericin B, voriconazole, and fluconazole (Table 2). Serum GM positivity was detected in 5/84 patients (6%) in the posaconazole group and in 5/34 patients (15%) in the control group (p=0.149). BAL GM positivity was detected in 4/15 patients (27%) in the control group and in 6/11 patients (55%) in the posaconazole group (p=0.227). However, 15/34 patients (44%) required the BAL procedure in the control group and 11/84 patients (13%) required this procedure in the posaconazole group (p<0.001). 

There was no mortality within 3 months of the completion of chemotherapy cycles among the AML patients with posaconazole prophylaxis. However, 18/34 patients (53%) in the control group died within 3 months of completion of their chemotherapy cycles. The 3-month mortality rate was significantly higher in control group (p<0.001).

## Discussion

Antifungal prophylaxis in hematology patients is important and reduces the use of antifungal therapy for suspected or proven IFIs, total mortality, and fungal infection-related mortality and minimizes the costs of management of either suspected or proven IFIs [15].

This study showed that antifungal prophylaxis with posaconazole significantly reduced IFIs and the need for antifungal treatment. Several recent studies supported the finding that posaconazole prophylaxis reduces the incidence of IFIs and invasive aspergillus in patients with AML/MDS or hematopoietic cell transplantation recipients when tested against comparable antifungal agents [16,17,18]. Prophylactic posaconazole was associated with statistically significantly fewer febrile days, shorter duration of hospitalization, and longer fungal-free survival; however, overall and attributable mortality did not differ [19]. In a study of 424 AML or MDS patients by Cho et al. [20], 140 received posaconazole and 284 received fluconazole prophylaxis. Fungal infection-free survival was significantly higher in the posaconazole group (74.7% vs. 87.1%, p=0.028). Investigators in Singapore created a network meta-analysis of randomized controlled trials evaluating posaconazole, concluding that it significantly reduced all-cause deaths compared to a fluconazole and itraconazole solution [21].

In patients receiving mold-active systemic antifungal prophylaxis with posaconazole, breakthrough IFIs occurred in 7.5% of patients [22]. Breakthrough infections are a major problem in patients receiving long-term prophylaxis [23]. Hoenigl et al. [24] proposed that GM testing is a useful diagnostic method for diagnosing breakthrough invasive aspergillosis in patients receiving mold-active prophylaxis and empirical therapy. In the study by Auberger et al. [25], breakthrough IFIs due to non-*Aspergillus* species, especially *Mucorales* spp., were noticed in a considerable proportion of patients at a high risk for IFIs receiving posaconazole prophylaxis. Bose et al. [26] reported that life-threatening *Fusarium* spp. infection may occur in immunocompromised patients despite prophylactic posaconazole.

It is assumed that azole-resistance could become a major problem in the future. Hamprecht et al. reported the first culture-proven case of invasive aspergillosis caused by azole-resistant *Aspergillus fumigatus* in a patient with AML in Germany, and this aspergillosis presented as a breakthrough infection under posaconazole prophylaxis [15]. Data from previous studies indicated that posaconazole is well tolerated, even following long-term administration. Several studies have shown that the most commonly reported adverse events were fever, nausea, diarrhea, vomiting, and headache [1,4,27,28,29]. In our study, posaconazole was discontinued within 7-14 days in 9/84 patients (11%) patients due to mucositis and diarrhea after chemotherapy. In our experience, prophylactic antifungal treatment is infrequently interrupted due to mucositis. Girmenia et al. [30] reported that posaconazole suspension might be used without the stringent need for monitoring plasma posaconazole concentrations in patients without diarrhea.

BAL GM has been recently explored as an additional method to diagnose invasive pulmonary aspergillosis. In those studies, the sensitivity of detection ranged from 57% to 88% and the specificity ranged from 87% to 95.8% [31]. In this study, there was no difference for serum and BAL GM positivity between the two groups. We found similar GM positivity within the two groups. We think that the low number of patients in the control group could be responsible for this result. On the other hand, it was shown that prophylaxis with posaconazole negatively affected GM test performance. It was shown that the serum GM test was unreliable in asymptomatic patients under anti-mold prophylaxis [32,33,34]. Previous exposure to antifungal agents should be considered when interpreting GM results.

### Study Limitations

The present study has some limitations. First, it is a retrospective study. Second, our control group was historical with a small sample size of controls, which was not matched numerically with the posaconazole prophylaxis group even at the minimum required optimal ratio of 1:1 to ensure reliable statistical analysis. Third, we did not measure plasma posaconazole levels. Finally, our study is a single-center study. In spite of these limitations of our study, we think that our results demonstrate the advantage of posaconazole prophylaxis in a real-life setting.

## Conclusion

This study showed that antifungal prophylaxis with a second-generation azole (posaconazole) can significantly reduce the need for antifungal treatment without the risk of increasing the rate of adverse events.

## Figures and Tables

**Table 1:**
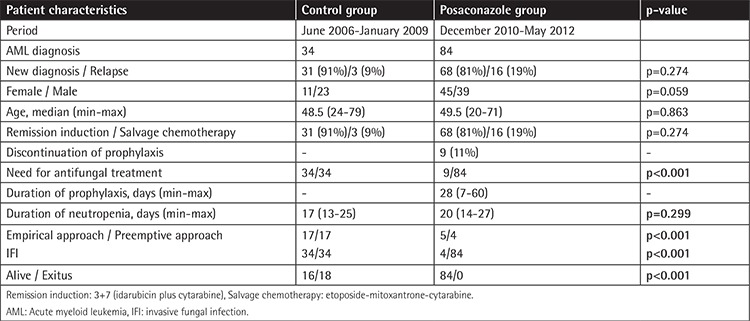
Characteristics of the patients and controls.

**Table 2:**
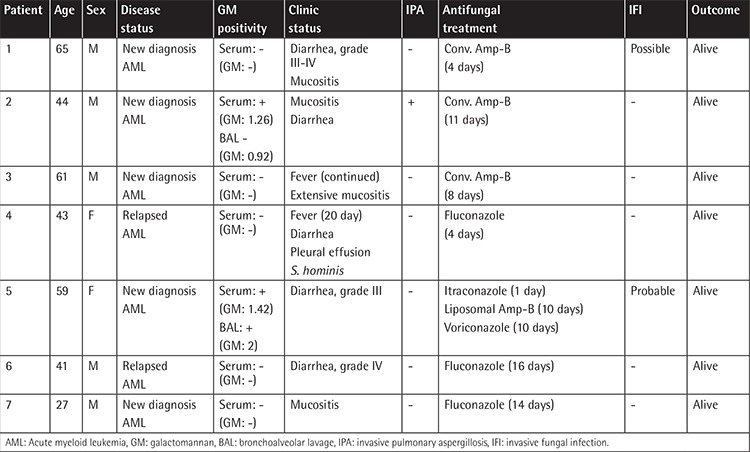
The clinical characteristics of seven patients with mucositis under posaconazole prophylaxis.
